# Self-Assembled Porous-Reinforcement Microstructure-Based Flexible Triboelectric Patch for Remote Healthcare

**DOI:** 10.1007/s40820-023-01081-x

**Published:** 2023-04-18

**Authors:** Hao Lei, Haifeng Ji, Xiaohan Liu, Bohan Lu, Linjie Xie, Eng Gee Lim, Xin Tu, Yina Liu, Peixuan Zhang, Chun Zhao, Xuhui Sun, Zhen Wen

**Affiliations:** 1 Institute of Functional Nano and Soft Materials (FUNSOM), Joint International Research Laboratory of Carbon-Based Functional Materials and Devices, Soochow University, Suzhou, 215123 People’s Republic of China; 2 Department of Electrical and Electronic Engineering, School of Advanced Technology, Xi’an Jiaotong-Liverpool University, Suzhou, 215123 People’s Republic of China; 3 Department of Applied Mathematics, School of Mathematics and Physics, Xi’an Jiaotong-Liverpool University, Suzhou, 215123 People’s Republic of China; 4 Department of Electrical and Electronic Engineering, University of Liverpool, Liverpool, L693GJ UK; 5 Jiangsu Key Laboratory for Carbon-Based Functional Materials and Devices, Soochow University, Suzhou, 215123 Jiangsu People’s Republic of China

**Keywords:** Pressure sensor, Triboelectric nanogenerator, Porous dielectric layer, Physiological signals, Internet of Healthcare

## Abstract

Realizing real-time monitoring of physiological signals is vital for preventing and treating chronic diseases in elderly individuals. However, wearable sensors with low power consumption and high sensitivity to both weak physiological signals and large mechanical stimuli remain challenges. Here, a flexible triboelectric patch (FTEP) based on porous-reinforcement microstructures for remote health monitoring has been reported. The porous-reinforcement microstructure is constructed by the self-assembly of silicone rubber adhering to the porous framework of the PU sponge. The mechanical properties of the FTEP can be regulated by the concentrations of silicone rubber dilution. For pressure sensing, its sensitivity can be effectively improved fivefold compared to the device with a solid dielectric layer, reaching 5.93 kPa^−1^ under a pressure range of 0–5 kPa. In addition, the FTEP has a wide detection range up to 50 kPa with a sensitivity of 0.21 kPa^−1^. The porous microstructure makes the FTEP ultra-sensitive to external pressure, and the reinforcements endow the device with a greater deformation limit in a wide detection range. Finally, a novel concept of the wearable Internet of Healthcare (IoH) system for real-time physiological signal monitoring has been proposed, which could provide real-time physiological information for ambulatory personalized healthcare monitoring.

**Supplementary Information:**

The online version contains supplementary material available at 10.1007/s40820-023-01081-x.

## Highlights

- The porous-reinforcement microstructure is constructed by the self-assembly of silicone rubber adhering to the porous framework of the PU sponge.
- With the excellent performance both for tiny pressure and large mechanical stimuli, the flexible triboelectric patch can be used to monitor pulse wave and plantar pressure.
- A remote healthcare system for real-time physiological signal monitoring is proposed.

**Supplementary Information:**

The online version contains supplementary material available at 10.1007/s40820-023-01081-x.

## Graphical abstract

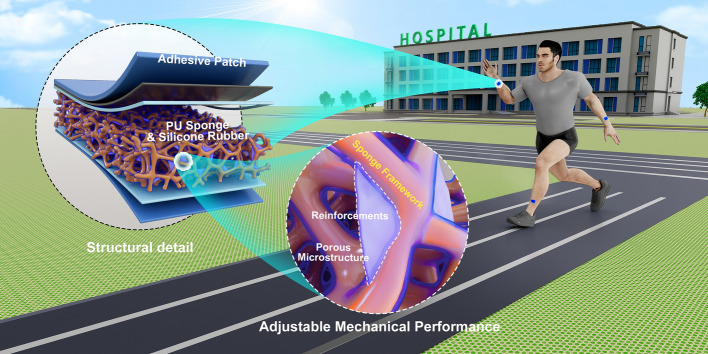

## Introduction

The Internet of Healthcare (IoH), which can upload users' physiological and behavioral data to healthcare centers for analysis and diagnosis with the help of wearable devices, is important for preventing and controlling chronic diseases common in elderly individuals [1–3]. Pressure signals, such as pulse waves, plantar pressure and joint angle, are the most basic information in daily activities that can reflect the health-related information of users [4, 5]. Highly sensitive wearable pressure sensors are therefore the basic and critical component of the IoH system [6, 7]. Generally, sensor notes are the primary source of high-power consumption in a typical Internet of Things (IoT). However, for an IoH system, real-time monitoring of the users' physiological signals over a long period is the most basic requirement, requiring wearable pressure sensors to be ultralow or even have zero power consumption [8–10].

The birth of triboelectric nanogenerators (TENGs), based on the coupling effect of triboelectrification and electrostatic induction, brings an effective strategy to achieve self-powered sensing [11, 12]. It has been extensively developed in recent years due to its natural advantages in terms of a wide range of material choices and the absence of electromagnetic interference [13–15]. Optimizing the mechanical properties of TENG-based sensors by building micro- and nano-structures is considered an effective way to improve sensing performance [16, 17]. Generally, triboelectric pressure sensors can be regarded as an electrical model of several capacitors connected in series [18, 19]. To enhance the sensitivity, a larger change in total capacitance at the same pressure is needed. Previous studies have demonstrated that building microstructures on the surface of the triboelectric layer can effectively amplify the capacitance variation between the top electrode and the dielectric layer and thus enhance the sensitivity of the device [20–22]. In addition, for the capacitance between the dielectric layer and the bottom electrode, building porous structures inside the dielectric layer has been proven to be an effective strategy to enhance the sensing performance [23–27]. However, although a large porous size could effectively improve the sensitivity of the triboelectric pressure sensor, the detection range of the sensor would be significantly shortened [28, 29]. How to balance the contradiction between sensitivity and detection range under high pressure is an urgent problem to be solved.

In this work, a highly sensitive flexible triboelectric patch (FTEP) with zero power consumption for remote health monitoring has been reported. The electromechanical coupling relationship of the proposed sensor with the porous-reinforcement microstructure has been established. The mechanical properties of the porous dielectric layer are regulated by introducing a porous sponge framework and silicone rubber reinforcements. Benefiting from the porous microstructures of the PU sponge framework, the sensitivity of the proposed FTEP can effectively be improved fivefold compared to devices with a solid triboelectric layer, reaching 5.93 kPa^−1^ under a pressure range of 0–5 kPa. In addition, the FTEP holds a high sensitivity of 0.21 kPa^−1^ even at a high pressure of 50 kPa. Moreover, the device possesses an ultrafast response time of 15 ms and an ultralow detection limit of 150 mPa. It has been successfully used to monitor some weak physiological signals, such as pulse waves, breathing rates and eye blinking, and also some large pressure signals, such as plantar pressure. Finally, a novel concept of the wearable IoH system for pulse wave detection consisting of an FTEP and signal processing and transmitting circuits has been demonstrated.

## Experimental Section

### Materials

The graphene ink was purchased from XFNANO Inc. Ethyl acetate was purchased from Sigma Aldrich. Ecoflex-00–30 was purchased from Dow Chemical Company. All materials and regents were used as received without further purification.

### Porous Dielectric Layer Preparation

First, a laser cutter was used to cut the PU sponge into 1.5 cm × 1.5 cm squares. Then, the sponges were soaked in ethanol and deionized water 3 times to clear the dust on the surface of the sponges and inside the pore. Then, the PU sponges were blown with nitrogen gas and set aside. Next, silicone rubber (Ecoflex-00–30) components A and B are mixed at a mass ratio of 1:1. Then, according to the volume ratio, the mixture was poured into an organic solution of ethyl acetate to form a uniform dilution of silicone rubber. Finally, the prepared sponges were placed into the silicone rubber dilution solution to fully absorb the dilution solution and then placed into the oven at 80 °C for 30 min. After solidifying in the oven, a negative triboelectric layer with a porous and reinforcement microstructure was obtained.

### Fabrication of FTEP

Graphene was chosen as the electrode, and another triboelectric layer was fabricated by printing graphene ink onto a PET substrate. More specifically, a commercial graphene paste was printed on a PET film with manual screen printing. The mesh number of the screen frame is 200. The film is then placed into a baking oven for curing of the printed graphene ink. The printed film is cured at 120 °C for 30 min in order that the graphene ink is fully dried. The other side of the PET substrate was adhered to aluminum foil by 3 M double-sided tape as the shielding layer. Double-sided adhesive is applied around the dielectric layer to form an air gap layer. Finally, adhesive fiber as the package layer wrapped the whole device.

### Characterizations

All scanning electron microscopy (SEM) images were taken by a Carl Zeiss Supra 55. The open-circuit voltage (*V*_oc_) and the short-circuit current (*I*_sc_) were measured by a Keithley 6514 electrometer. The Finite Element Analysis (FEA) was realized by SolidWorks and COMSOL Multiphysics. The sensing performance of the device to pressure signals was measured by Chatillon DFS force measurement (AMETEK Co., Ltd.) and Keithley 6514 electrometer. The software platform based on LabVIEW can realize real-time data acquisition control and analysis.

## Results and Discussion

### Device Structure and Working Mechanism

**Fig. 1:**
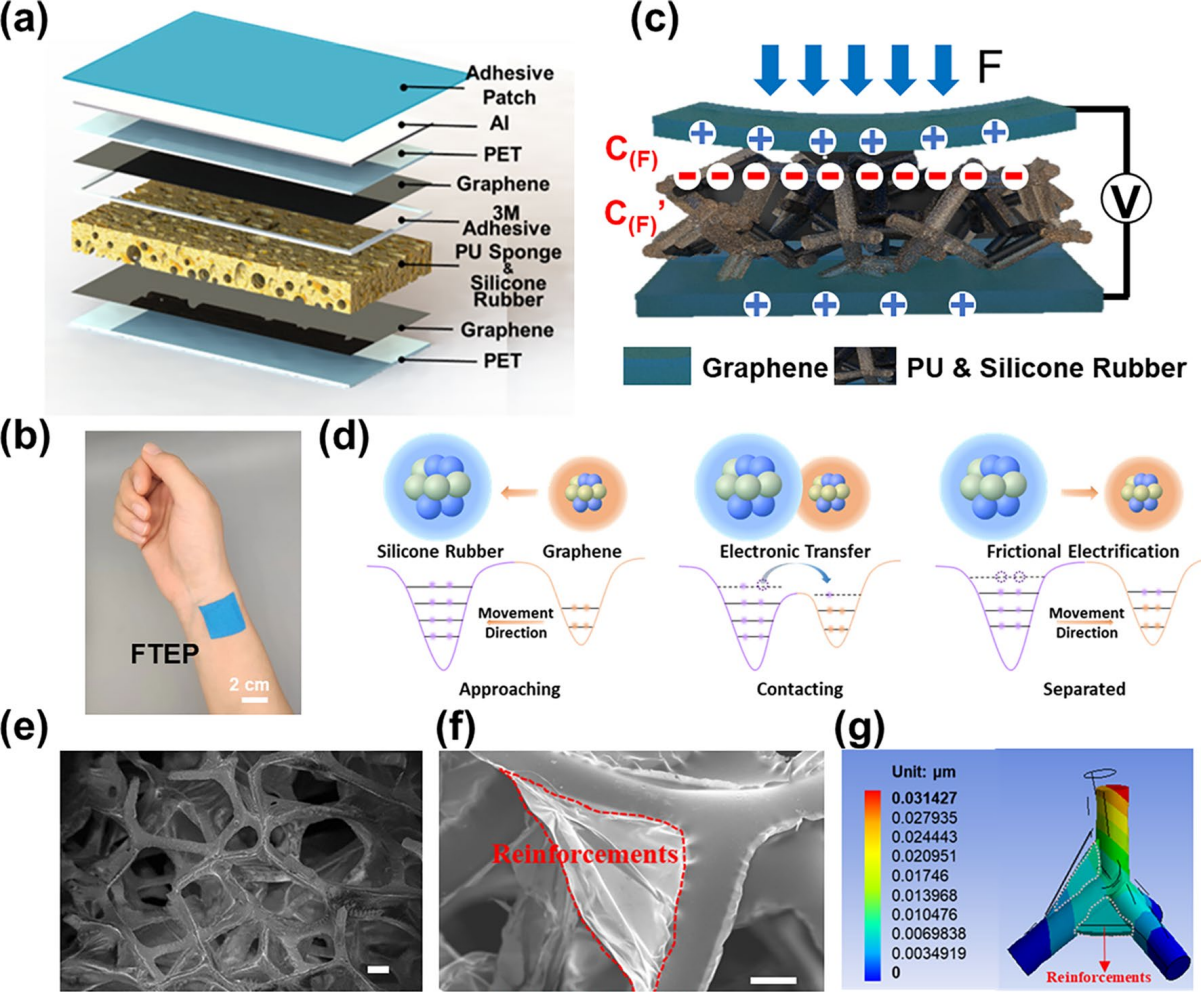
Structural and mechanism model of the flexible triboelectric patch (FTEP). Schematic illustrations of (**a**) structure and (**b**) equivalent electrical model of the FTEP. (**c**) Picture of the FTEP attached to human skin. (**d**) Atomic-scale electron cloud potential well model to describe the contact-separate processing of FTEP. SEM image of the pure PU sponge (**e**) without silicone rubber dilution and (**f**) with 30% silicone rubber dilution (scale bars, 100 μm). (**g**) FEA result of the PU sponge with 30% silicone rubber dilution

The FTEP consists of an adhesive patch as the package layer, an aluminum shielding layer, a pair of graphene electrodes, a double-sided adhesive as the spacer and a porous dielectric film, as shown in Fig. 1a. Aiming at strengthening the anti-oxidation capability of electrodes, graphene is selected as the conductive material to be printed on the PET substrate. Figure S1 shows the optical picture of conductive graphene ink and the SEM picture of graphene electrode after the printing is completed. The entire device is highly flexible, as shown in Fig. 1b; thus, the acquisition of physiological signals can be accomplished by attaching the patch to a specific part of the body. The physical model of the FTEP is illustrated in Fig. 1c. Under the pressure of an external force, the upper graphene electrode comes into contact with the silicone rubber on the surface of the sponge framework. Due to the coupling effect of the triboelectrification and the electrostatic induction, the FTEP can convert the mechanical pressure into an electricity signal [30]. The process of contact electrification can be well described by an atomic-scale electron cloud potential well model between graphene and the silicone rubber layer (Fig. 1d and Note S1) [31, 32]. Figure S2 shows the electrical model of the FTEP in its working state. In the open-circuit status, the FTEP can be equated to two capacitors in series. Under certain pressure, the upper graphene electrode and the porous dielectric film deform simultaneously, resulting in a change in the potential difference between the two electrodes. The pressure signal can therefore be obtained in real time by monitoring the FTEP’s voltage change.

To improve the sensitivity and extend the detection range of the FTEP, porous-reinforcement microstructures are constructed in the dielectric layer. The porous dielectric film here was fabricated through a convenient and controllable method rather than using the conventional way, water-soluble salt as a template (Fig. S3). The PU sponge with a natural porous structure is chosen as the framework, which can be seen as a porous structure formed by a large number of tetrahedral skeletons connected end to end (Fig. 1e). Silicone rubber adheres to the framework to play the role of reinforcements. When the sponge framework is impregnated with different concentrations of silicone rubber solution, the silicone rubber enters the pores and fills the pore structure of the sponge. When the silicone rubber is cured and heated, the organic solvent is evaporated by the heat, leaving only the cured and cross-linked silicone rubber behind, as shown in Fig. S4. The final volume of silicone rubber left behind varies with different solution concentrations, resulting in porous dielectric layers with different mechanical performances. The size of the reinforcements can be easily adjusted by changing the ratio of solvent to silicone rubber, thus controllably modulating the mechanical properties of the porous dielectric film. Here, the dilution concentration is represented by the volume ratio of ethyl acetate to silicone rubber. This means that the greater the volume ratio is, the greater the dilution.

Figures 1f and S5 show the SEM image and optical magnifications of the porous dielectric layer at a dilution concentration of 30%. The cured silicone rubber content is small and mostly wrapped around the PU sponge framework, with only a tiny fraction of silicone rubber present in the pores. The silicone rubber could thicken the diameter of the PU sponge framework, and the small amount of silicone rubber in the pores acts as a reinforcement against deformation caused by external forces, resulting in enhanced mechanical properties. As shown in Fig. S6, finite element analysis (FEA) was used to verify the contribution of the porous microstructure to the mechanical properties of the porous dielectric layer. Smaller reinforcement bars are placed at the edges of the tetrahedral framework, which are used to simulate the small amount of silicone rubber present in the pores. It can be observed that the tetrahedral framework undergoes a large compressive deformation under external pressure as shown in Fig. 1g. In addition, the optical magnifications, SEM image and FEA result of the porous dielectric layer at a dilution concentration of 15% are shown in Fig. S7. By placing larger reinforcement bars around the edges of the tetrahedral framework to simulate the presence of silicone rubber in the pores, it can be found that the tetrahedral framework undergoes less compressive deformation under the same external pressure compared with the FEA results with 30% silicone rubber dilution. This is the reason that the compressive modulus of the porous dielectric layer increases significantly when the dilution concentration is changed from 30% to 15%.

### Sensing Performance of the FTEP

**Fig. 2:**
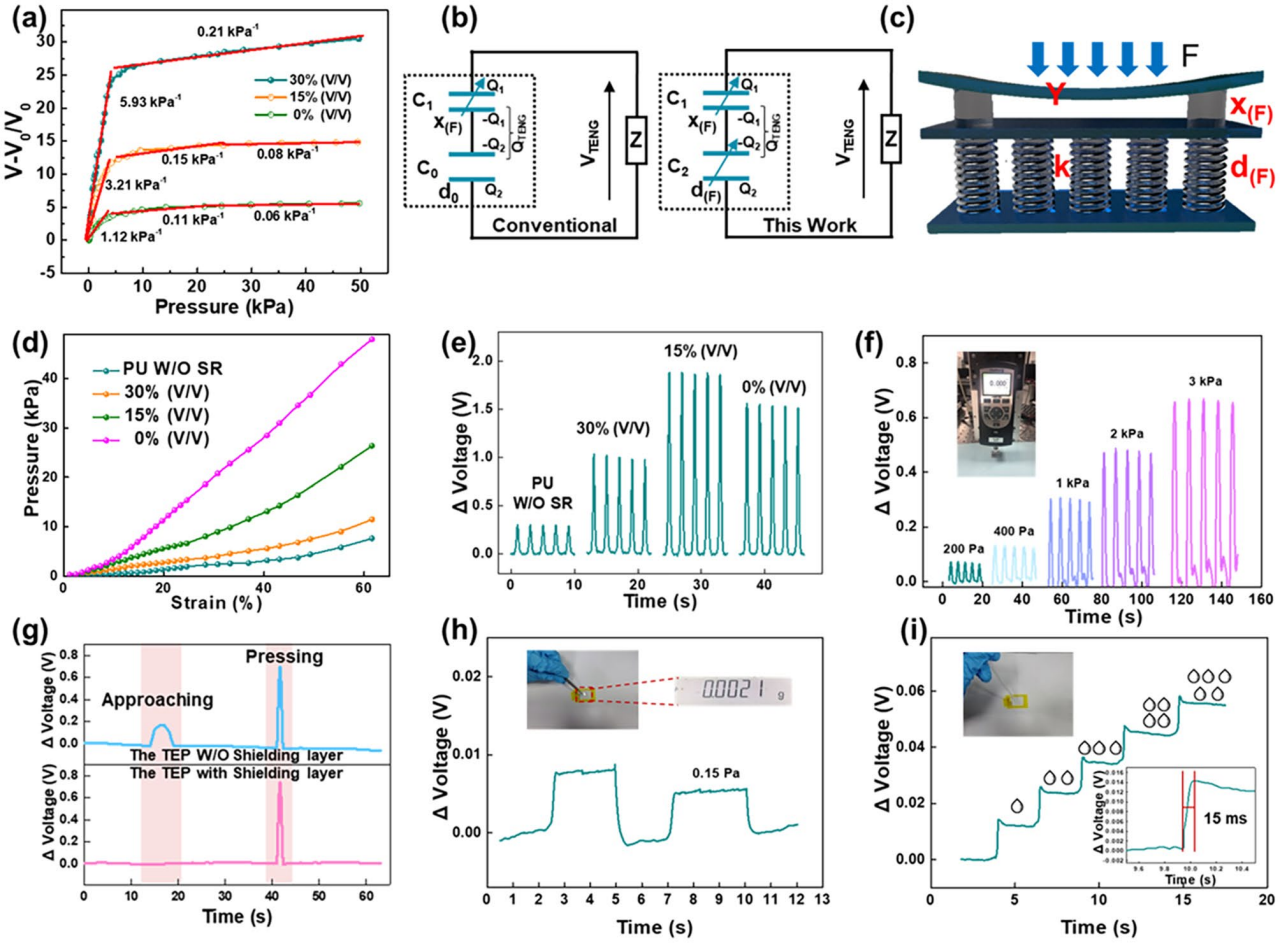
Pressure sensing performance and mechanical properties of the FTEP. (**a**) Sensitivity curves of the FTEP with various proportions of silicone rubber. (**b**) Equivalent capacitance models of the conventional device and FTEP. (**c**) Equivalent mechanical model of FTEP. (**d**) Stress‒strain curves of PU sponges with various proportions of silicone rubber. (**e**) Voltage curves of the FTEP with various proportions of silicone rubber under the same pressure. (**f**) Real-time response to vertically variable pressure. (**g**) Comparison of the real-time response to the approach and pressure of a human hand. (**h**) Voltage signals of the FTEP generated by a very fine piece of aluminum foil with 0.15 Pa. (**i**) Relative voltage change by loading several water droplets falling from a height of 2 cm one by one. The inset is the partial enlargement during three water droplets falling down

Figure 2a shows the sensitivity curves for the sensors consisting of porous dielectric layers with different dilution concentrations. It is clear that the sensitivity gradually increases with increasing dilution concentration in both the small and large pressure zones. The sensitivity of the proposed FTEP with a 30% dilution concentration can effectively be improved fivefold compared to the sensors with a solid triboelectric layer, reaching 5.93 kPa^−1^ in the 0–5 kPa detection region. When the pressure exceeds 5 kPa, the sensitivity of all three devices decreases substantially. However, the sensitivity of the proposed FTEP with a 30% dilution concentration, reaching 0.21 kPa^−1^, is still higher than that of the other two devices. Moreover, when the pressure exceeds 20 kPa, the sensitivities of the other two sensors decrease to below 0.1 kPa^−1^. For further comparison, the sensing performance of TENG-based pressure sensors with porous microstructure and surface microstructure in recent years has been summarized in Table S1. It can be found that the device with porous-reinforcement microstructure in this work has more advantages in achieving high sensitivity sensing with wide detection domain. The effect of the dilution concentration on the sensing performance can be summarized through the constructed electrical and mechanical models.

As shown in Fig. 2b, triboelectric pressure sensors can be regarded as an electrical model of two capacitors connected in series, which are the upper capacitor between the top electrode and the dielectric layer and the lower capacitor between the dielectric layer and bottom electrode. The upper capacitor has an air gap of *x*_(F)_, the capacitance is *C*_1,_ and the lower capacitor has a dielectric layer thickness of *d*_(F)_. For the conventional triboelectric sensor structure, to acquire high sensing performance, the upper capacitance *C*_1_ tends to be easily changed with the variation of the applied pressure. However, the lower capacitance *C*_0_ generally remains constant because the height of the lower part of the dielectric layer is generally difficult to change due to the limited preparation process. In this case, the sensitivity and detection range of the triboelectric sensor are limited by the mechanical properties of the upper part of the dielectric layer. Therefore, the modulation of the mechanical properties of the lower capacitor *C*_2_ of the dielectric layer is also worthy of attention. As shown in Fig. S8**,** the total voltage of these two capacitors in series is *V*_(F)_, which is determined by the capacitance value of the two capacitors (Note S2). When an external force is applied to the triboelectric sensor, the capacitance value of the two capacitors changes accordingly, resulting in a corresponding change in voltage. The relationship between the electrical potential difference *V*_(F)_ and the height of the gap layer and dielectric layer under the external force *F* can be obtained [33, 34]:

1 $$ V_{\left( F \right)} = \frac{1}{{\varepsilon_{0} A}}\left( {\frac{{{\text{Q1}}x_{\left( F \right)} }}{{\varepsilon_{1} }} + \frac{{{\text{Q2}}d_{\left( F \right)} }}{{\varepsilon_{2} }}} \right) $$

where ${\varepsilon }_{0}$, ${\varepsilon }_{1}$ and ${\varepsilon }_{2}$ are the vacuum permittivity, air permittivity and dielectric layer permittivity, respectively. ${\text{Q}}{1}$ and ${\text{Q}}{2}$ are the amounts of charges on the upper electrode and the lower electrode, respectively.

Figures 2c and S9 show the mechanical models of the FTEP. As the upper electrode is attached to the surface of the porous dielectric layer by double-sided adhesive, the upper electrode can be considered a simply supported beam model, which undergoes bending deformation when an external force *F* is applied to the FTEP. Additionally, the applied force can be transferred to the porous dielectric layer, causing it to undergo compressive deformation. As shown in Fig. 2c, the porous dielectric layer can be seen as a spring model with multiple parallel connections. The electrode distances of these two capacitors under the external force *F* are *x*_(F)_ and *d*_(F)_. Therefore, the potential difference *V*_(F)_ between the two electrodes of the FTEP under the external force *F* can be expressed as:

2 $$ V_{\left( F \right)} = \frac{{Q_{1} }}{{\varepsilon_{0} \varepsilon_{1} A}}\left( {x_{0} - \frac{FL3}{{48YI}}} \right) + \frac{{Q_{2} }}{{\varepsilon_{0} \varepsilon_{2} A}}\left( {d_{0} - \frac{F}{k}} \right) $$

where *L* is the length of the upper electrode. *Y* is the Young’s modulus of the upper electrode, and *I* is the moment of inertia. *k* is the equivalent stiffness coefficient of the parallel springs system. The above equation shows that the potential difference of the FTEP is closely related to the mechanical properties of the air gap and the dielectric layer. A high sensitivity means that the output voltage of the FTEP varies more under the same external force. According to the analysis of the factors influencing the sensitivity in Note S2, it can be concluded that the lower Young’s modulus of the upper electrode and equivalent stiffness coefficient of the porous dielectric layer favor improving sensitivity.

In the low-pressure region, the upper electrode deforms into the air cavity and gradually touches the dielectric layer. The voltage change is mainly due to the change in capacitance of *C*_1_ in this situation. At the same time, pressure is transferred to the dielectric layer through the spacer layer, causing a small compressive deformation of the dielectric layer. These two reasons together contribute to the high sensitivity of the sensors in the small pressure region. In the large pressure region (> 5 kPa), where the upper electrode film has already been in close contact with the dielectric layer, the deformation mainly arises from further compression of the porous dielectric layer. As the dielectric layer of the 30% dilution retains partial pores in the PU sponge framework, it still has some compression properties in the large pressure range relative to the devices with 0% and 15% dilution concentrations, allowing the sensor to remain highly sensitive in the large pressure region.

To sum up, the higher sensitivity at low pressure of the device with 30% dilution concentrations is attributed to the deformation of porous-reinforcement microstructures at low pressure, which leads to the larger voltage variation comparing with the sensors with 0% and 15% dilution concentrations. In addition, the height variation of dielectric layer approaches saturation when the pressure is larger than 5 kPa, resulting in a much slowly change of the output voltage. However, the sensitivity of the device with 30% dilution concentrations under large pressure region is still higher than the sensors with other two kinds of dilution concentrations, benefiting from the presence of partial pores. Moreover, the presence of the reinforcements keeps the porous dielectric layer from reaching its deformation limit at a relatively small pressure, which endows the FTEP with a wide detection range of 50 kPa.

The mechanical performance of the porous-reinforcements microstructures-based dielectric layer has been investigated to verify the above illustration. Figure 2d shows the stress‒strain curves of porous dielectric layers with different concentrations of silicone rubber solution. The equivalent stiffness coefficient of the 30% silicone rubber dilution does not change much compared to the pure PU sponge. In contrast, when the dilution concentration is reduced to 15%, a substantial increase in the equivalent stiffness coefficient of the porous dielectric layer occurs. At the same pressure, the dielectric layer with 30% silicone rubber dilution undergoes greater deformation, resulting in a greater change in the potential difference. However, the equivalent stiffness coefficient of the device with 30% silicone rubber dilution is still slightly higher than that of the pure sponge due to the presence of the reinforcements, which endows the sensor with a wide detection range.

In addition to the mechanical properties, the output voltage of the FTEP also plays an important role in judging the sensing performance, as it can determine the signal–noise ratio. Figure 2e shows various concentrations of silicone rubber dilution. The output voltage of the sensors with various concentrations of silicone rubber dilution gradually increases as the dilution concentration decreases. However, when the dilution concentration is reduced to 0, the output voltage is slightly lower than that of the 15% device. Silicone rubber is much more electronegative than PU sponge, and it acquires more charge during the contact-separation process with the graphene electrode. As a result, the output voltage of a porous dielectric layer with a 30% dilution concentration increases compared to a pure PU sponge. As the dilution concentration decreases, the silicone rubber content in the pores of the sponge increases. Thus, the contact area between the silicone rubber on the upper surface of the PU sponge and the electrode becomes larger during the contact-separation process, leading to a further increase in the output voltage. However, when the dilution concentration is less than 15%, the internal pores of the sponge are basically filled, and the contact area does not continue to increase at the same pressure. At the same time, the reduction in internal pores leads to a change in the effective permittivity of the dielectric layer, which reduces the output voltage. Although the output voltage of the 30% dilution concentration device is slightly lower than that of the 15% dilution concentration device, its mechanical properties are much better. For a triboelectric pressure sensor, the need for high sensitivity is much stronger than the output voltage due to the existence of the amplifier circuit. Therefore, the sensor patch with the 30% dilution concentration dielectric layer was chosen for the following demonstration.

To examine the pressure sensing of the FTEP, the different output voltages of the FTEP under different pressures were measured. As the pressure increased from 0.2 to 3 kPa, the output voltage increased from 0.1 to 0.7 V, demonstrating the excellent detection resolution (Fig. 2f). The movement of external objects can cause extensive interference to the triboelectric sensor output signal. This work applies a shielding layer to the device surface realizing the shielding of external interference signals. Figure 2g shows the results of an FTEP with/without a shielding layer when a finger is approaching and pressing the device. The shielding layer is able to shield the interference signal effectively when the finger is approaching. The pressures that carry physiological signals from the human body are very weak, so the sensor needs to have excellent sensing performance even under tiny pressures (Movie S1). Figure 2h shows the detection limitation of the FTEP. When an aluminum foil weighing 2.1 mg (square with 3.7 mm sides) is dropped on the FTEP, the equivalent pressure is approximately 0.15 Pa, and the sensor can output a voltage signal of ~ 8 mV. In addition, the sensor can monitor the pressure changes caused by water drops falling drop by drop on the sensor surface, as shown in Fig. 2i. The inset figure illustrates the rapid response time of the sensor to small changes in external pressure, allowing real-time monitoring of pressure within 15 ms. In addition, the FTEP can generate a noticeable voltage change of 0.1 V by air flow from the compressed elastic ball, demonstrating its potential as a respiratory sensor to monitor airflow (Fig. S10a). The device also shows excellent repeatability and stability after 4000 loading/unloading cycles (Fig. S10b). To evaluate the dynamic pressure sensing performance of the FTEP, the short-circuit current under variable pressure and different frequencies was measured, as illustrated in Fig. S11. The results indicate that the current increases steadily with the elevated frequency under the same applied force, which proves that the FTEP can detect the loading frequency effectively. The results also demonstrate that FTEP enables the accurate detection of high-frequency vibration signals caused by sound.

### Applications of the FTEP in Remote Healthcare

**Fig. 3:**
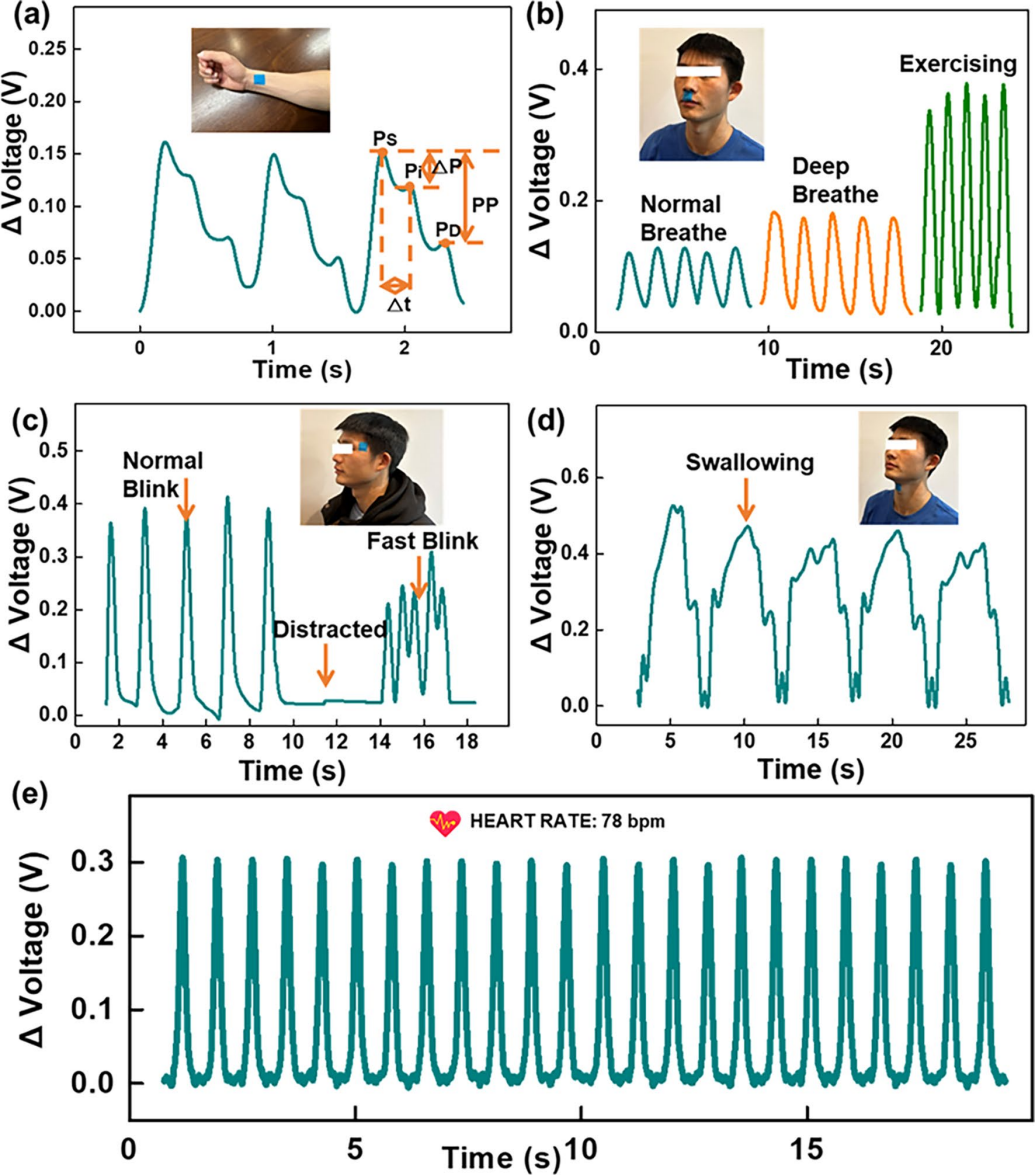
Real-time dynamics of FTEP in physiological signal monitoring. (**a**) Pulse wave of a volunteer detected by FTEP. (**b**) Respiratory signals include respiratory rate and intensity monitored by the FTEP. (**c**) Blink frequency signals monitored by the FTEP on the corner of the eye. (**d**) Swallowing rate monitored by the FTEP on the throat. (**e**) Heart rate monitored by FTEP on the chest

The as-prepared FTEP for real-time monitoring of common human physiological signals has been demonstrated, aiming to verify the potential of the FTEP for applications in IoH systems. As shown in Fig. 3a, the pulse wave signal can be successfully monitored in real time by attaching the FTEP to the volunteer’s wrist. It can clearly detect the characteristic peaks of the pulse wave, providing the basis for accurate extraction of parameters related to cardiovascular health (Movie S2). More specifically, there are three critical feature point of pulse waves, the peak of the advancing wave (*P*_s_), the peak of the reflected wave (*P*_i_), and the peak of the dicrotic wave (*P*_d_). These peaks can be used to further calculate the detail feature parameters for artery evaluating. Parameters such as diastolic blood pressure, systolic blood pressure and blood flow velocity shown in the diagram can be used to accurately predict the user's cardiovascular status through cloud-based algorithms, enabling early warning of sudden cardiovascular disease.

The FTEP can also be attached under the user's nose so that the respiratory status can be monitored in real time by detecting the pressure change caused by the airflow on the sensor surface during breathing. As shown in Fig. 3b, the waveform of the user's normal, deep and postexercise breathing can be clearly distinguished by detecting the amplitude and frequency of the output voltage of the FTEP. Figure 3c demonstrates the real-time monitoring of the user's blink frequency. The system can determine how often the user's dull gaze occurs, thus providing early warning of diseases such as dementia. Figure 3d shows an FTEP attached to the user's throat that can accurately monitor the user's swallowing movements, which could provide data to support the monitoring of the user’s eating behavior. In addition, attaching the FTEP on the chest also allows for real-time monitoring of the user's heart rate. Figure 3e shows the volunteer's regular heart rate during the experiment, and the result is 78 bpm. Owing to the wide detection range of the FTEP, it can be used not only for the monitoring of the above-mentioned tiny pressure signals, but also for the monitoring of large pressure such as the sitting state and sedentary as demonstrated in Fig. S12a. In addition, because the sensor still has high sensitivity in the large pressure region, the FTEP can clearly distinguish the weight signals of the motorcycle and the human body as shown in Fig. S12b. Benefited from the 2 × 2 sensor’s high sensitivity, the shifts in human body gravity center make the plantar pressure distribution clearly detectable as shown in Fig. S13.

**Fig. 4:**
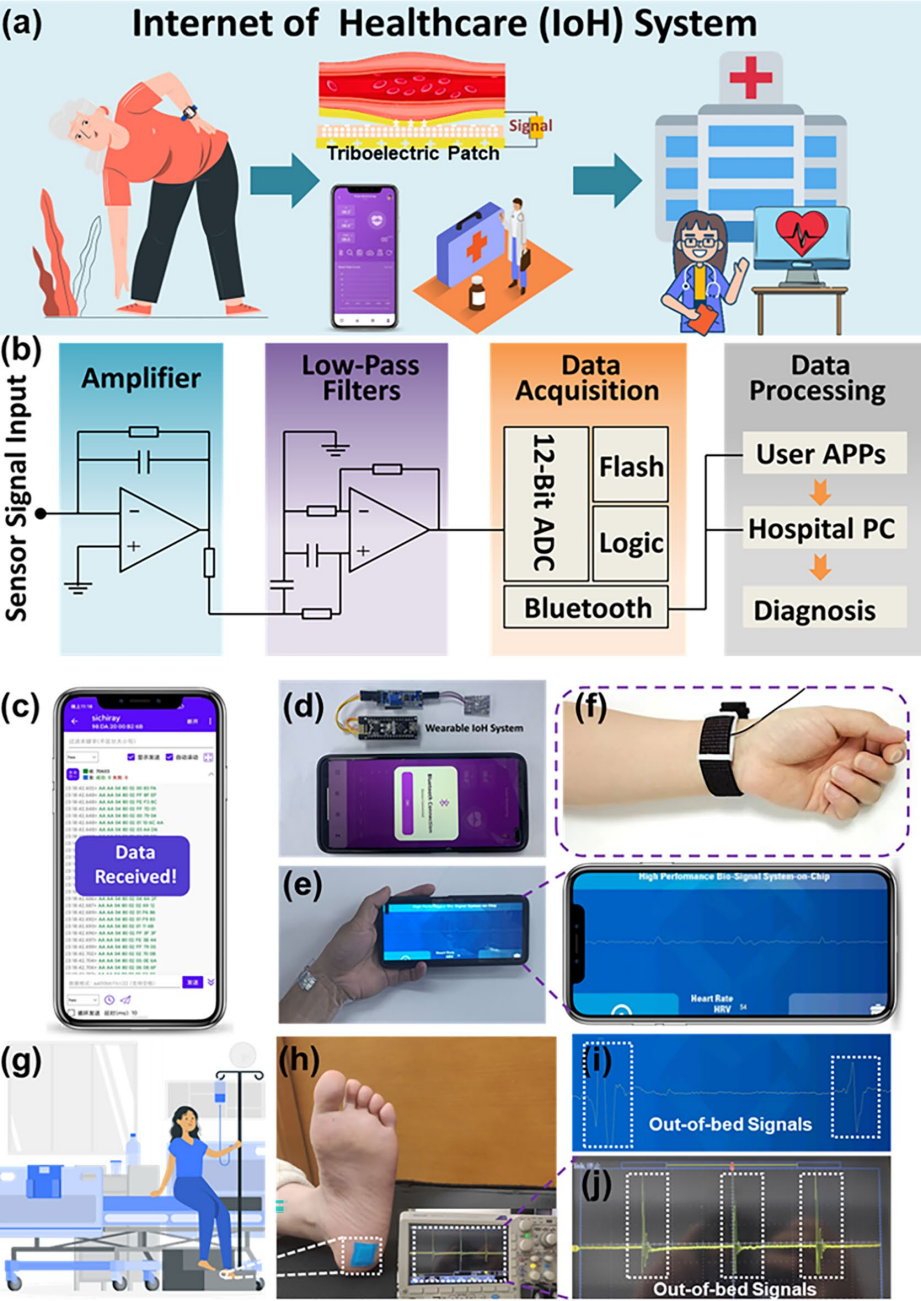
Demonstration of the IoH system and its application in ambulatory personalized healthcare monitoring. (**a**) Systematic configuration of the FTEP-based Internet of Healthcare (IoH) system in which the collected signals can be wirelessly transmitted to a cellphone and hospital data centers. (**b**) A schematic diagram of the IoH system composed of four components of signal amplifier, low-pass filters, data acquisition and data processing. (**c**) A photograph of the Bluetooth data interface while receiving the pulse wave signal. (**d**) A photograph of the IoH system for users. (**e**) Pulse wave of the volunteer displayed on a cellphone interface using a customized app program. (**f**) A photograph of the FTEP attached to the strap for the convenience of users. (**g**) A demonstration of FTEP as wearable electronics to monitor the out-of-bed status of patients or elderly individuals. (**h**) A photograph of the FTEP attached to the heel to monitor out-of-bed status. (**i**) Received out-of-bed results in the nurse’s APP from FTEP. (**j**) Output signal during out-of-bed status detected by the oscilloscope

Given the compelling features of the FTEP, an IoH system for the real-time monitoring of physiological signals has been established. As shown in Fig. 4a, the core of an IoH system consists of a highly sensitive health monitoring sensor module, a data display module for users, and a diagnosis module for medical staff. For example, the highly sensitive FTEP can be worn on the user's wrist by integrating it with a watch, converting the weak mechanical energy of the arterial beat into a recognizable electrical signal. Afterward, the pulse wave can be captured, and the waveform signal is capable to be sent to the user's mobile phone and the medical staff’s computer in real time via wireless communication. The triboelectric sensor is typically a high-impedance electronic device, and due to the impedance mismatch, it usually exhibits a very low effective signal when connected directly to external circuits. Therefore, before ADC is performed on the TENG output signal, its signal needs to be amplified and filtered and finally sent to the user’s phone via Bluetooth for data processing and calculation, whose data transmission process is shown in Fig. 4b. Figure 4c shows the Bluetooth data reception interface of the user’s App when the phone successfully receives sensor data that have been converted to 16-bit strings via ADC. Figure 4d shows the whole wearable IoH system based on FTEP, which can be easily applied to the skin by an adhesive patch. Figure 4e shows the real-time monitoring interface for measuring the user’s pulse wave for healthcare. The tiny pressure signal can be wirelessly transmitted to a mobile phone and displayed on a customized app program (Movie S3). For easy wear and effective adjustment of the preload force, the sensor can be attached to the strap, as shown in Fig. 4f, and the strap can be adjusted easily, providing a more comfortable measurement experience.

In addition to monitoring small pressure signals, the FTEP can also be attached to the heel for large pressure real-time monitoring, such as out-of-bed warnings, as shown in Fig. 4g, h. Figure 4i shows the App interface when a patient is getting out of bed, and the pressure signal can be wirelessly transmitted to a mobile phone and displayed on a customized App program. Due to the impedance mismatch, the signal of the triboelectric pressure sensor is generally difficult to monitor directly by the oscilloscope. Here, through the proposed signal processing circuit, the oscilloscope can clearly detect the signal of human plantar pressure to realize the out-of-bed warning for patients or the elderly (Fig. 4j). Additionally, tiny physiological signals such as pulse waves can also be detected with a high signal–noise ratio, as illustrated in Fig. S14. It shows the high efficiency of the data processing circuit to extract and amplify the weak signals.

## Conclusions

In summary, this work reports a highly sensitive triboelectric patch for remote health monitoring and a feasible method for preparing a porous-reinforcement microstructure-based triboelectric layer. With the aid of the porous PU sponge’s self-adsorption characteristics to low-viscosity solutions, the volume of polymer remaining on the sponge framework can be controlled by adjusting the volume ratio of polymer to organic solvent. Hence, the porous triboelectric layer’s mechanical properties can be further manipulated. Moreover, the electromechanical coupling relationship of the FTEP has been established, and the effect of each functional layer on the sensing performance of the device has also been explored. Owing to the outstanding mechanical performance of the porous-reinforcement microstructure-based triboelectric layer, the sensitivity of the proposed FTEP can effectively be improved fivefold compared to devices with a solid triboelectric layer, reaching 5.93 kPa^−1^ under a pressure range of 0–5 kPa. The proposed device possesses a high sensitivity of 0.21 kPa^−1^ even in the high-pressure region to 50 kPa, which is mainly attributed to the fact that the porous triboelectric layer with reinforcements can still undergo some deformation under high pressures. In addition, FTEP has an ultrafast response time of 15 ms and an ultralow detection limit of 150 mPa. Therefore, it has been successfully used to monitor some weak physiological signals, such as pulse waves, breathing rates, eye blinking and some large pressure signals, such as planter pressure. Finally, a novel concept of a wearable IoH system for pulse wave detection consisting of an FTEP and signal processing and transmitting circuits has been proposed. The wearable IoH system could provide direct and real-time pulse waves to the medical side for ambulatory personalized cardiovascular disease monitoring. Above all, the developed triboelectric patch sensor-enabled wireless biomonitoring system is a solid step for the FTEP toward the Internet of Healthcare era.

### Supplementary Information

Below is the link to the electronic supplementary material.

Supplementary file1 (AVI 6845 kb)

Supplementary file2 (AVI 1806 kb)

Supplementary file3 (AVI 2449 kb)

Supplementary file4 (PDF 1183 kb)
