# Supplementary material for: Self-Assembled Porous-Reinforcement Microstructure-Based Flexible Triboelectric Patch for Remote Healthcare
Source: Nanomicro Lett. 2023 Apr 18;15:109. doi: 10.1007/s40820-023-01081-x (PMC10113410; doi:10.1007/s40820-023-01081-x)
Supplement: Supplementary file 4 — Supplementary file4 (PDF 1183 kb) [file 40820_2023_1081_MOESM4_ESM.pdf]

Supporting Information for

Self-Assembled Porous-Reinforcement Microstructure-Based Flexible Triboelectric Patch for Remote Healthcare

Hao Lei[1, 2, 4, #], Haifeng Ji[1, #], Xiaohan Liu[5], Bohan Lu[3, 4], Linjie Xie[2, 3], Eng Gee Lim[2], Xin Tu[4], Yina Liu[3], Peixuan Zhang[1], Chun Zhao[2, *], Xuhui Sun[1, 5, *] and Zhen Wen[1, *]

[1] Institute of Functional Nano and Soft Materials (FUNSOM), Joint International Research Laboratory of Carbon-Based Functional Materials and Devices, Soochow University, Suzhou 215123, P. R. China

[2] Department of Electrical and Electronic Engineering, School of Advanced Technology, Xi'an Jiaotong-Liverpool University, Suzhou 215123, P. R. China

[3] Department of Applied Mathematics, School of Mathematics and Physics, Xi'an Jiaotong-Liverpool University, Suzhou 215123, P. R. China

[4] Department of Electrical and Electronic Engineering, University of Liverpool, Liverpool L693GJ, United Kingdom

[5] Jiangsu Key Laboratory for Carbon-Based Functional Materials & Devices, Soochow University, Suzhou, 215123, Jiangsu, P. R. China

[#] H. Lei and H. Ji contributed equally to this work.

*Corresponding authors. E-mail: chun.zhao@xjtlu.edu.cn (C. Zhao); xhsun@suda.edu.cn (X. Sun); wenzhen2011@suda.edu.cn (Z. Wen)

Note S1 Working Mechanism of the Porous Flexible Triboelectric Patch (FTEP)

The electron clouds are separated as shown in Fig. 1d when the different charge affinity atoms in silicone rubber and graphene are far apart, and in this situation their electrons are confined in their atom orbits with high escaping energy barriers. Under a pressure on the FTEP, the atoms of these two materials gradually close to a distance shorter than the bonding length and eventually reach the repulsive force region. In this case, the electron clouds of silicone rubber and graphene strongly overlap, and the interatomic potential barrier decreases significantly. As a result, some electrons in graphene with higher energy can easily overcome the reduced potential barrier and therefore transfer to silicone rubber to achieve equilibrium. As the pressure decreasing, silicone rubber and graphene separate and the transferred electrons and holes remain as static charges on the surfaces.

From a macro perspective, when the pressure is applied on the FTEP, the upper electrode of graphene is in contact with silicone rubber and the charges will transfer from the graphene to the surface of silicone rubber due to the difference in

electronegativity. At the same time, the transferred charges trapped at the surface will quickly compensate each other, and no current flows through the external circuit (Fig. S2a). As the pressure decreasing, the upper electrode of graphene and silicone rubber separate from each other and the transferred charges will cause an electric potential between two electrodes by electrostatic induction to drive the current to flow through the external circuit (Fig. S2b). Once the pressure applied on the FTEP decreases to 0, the upper electrode and the dielectric layer are fully separated and there is no current flowing between two electrodes. At this time, the electric potential difference between two electrodes remains constant and this state is defined as the initial state of the open-circuit voltage measurement (Fig. S2c). The electrical model of the FTEP can be expressed as Fig. S2d.

From the Gauss theorem, the electric field strength in the air gap region E_1 is given by [S1]:

$$E_1 = \frac{Q_1}{\varepsilon_0 \varepsilon_1 A} \tag{S1}$$

The electric field strength E_2 in the dielectric region is given by:

$$E_2 = \frac{Q_2}{\varepsilon_0 \varepsilon_2 A} \tag{S2}$$

The voltage between the two electrodes $V_{(F)}$ can be given by:

$$V_{(F)} = E_1 \cdot x_{(F)} + E_2 \cdot d_{(F)} \tag{S3}$$

Substituting Eqs. (S1) and (S2) into Eq. (S3), we can obtain that:

$$V_{(F)} = \frac{1}{\varepsilon_0 A}\left(\frac{Q_1 x_{(F)}}{\varepsilon_1} + \frac{Q_2 d_{(F)}}{\varepsilon_2}\right) \tag{S4}$$

where Q_1 and Q_2 are the electrical charge in the upper electrode and bottom electrode. $x_{(F)}$ and $d_{(F)}$ are the distance from the upper electrode to the surface of dielectric layer, and surface of dielectric layer to the bottom electrode.

Note S2 Electrical and Mechanical Models for the FFTEP

From the perspective of electrical circuit, the FTEP can be seen as a model of two capacitors connected in series as shown in Fig. S8a-b. The capacitance between top electrode and the dielectric layer C_1 is given by [S2, S3]:

$$C_1 = \frac{\varepsilon_0 \varepsilon_1 A}{x_{(F)}} \tag{S5}$$

The capacitance between the dielectric layer and top electrode C_2 is given by:

$$C_2 = \frac{\varepsilon_0 \varepsilon_2 A}{d_{(F)}} \tag{S6}$$

The voltage between the two electrodes $V_{(F)}$ can be given by:

$$V_{(F)} = V_1 + V_2 \tag{S7}$$

$$V_1 = \frac{Q_1}{C_1} \tag{S8}$$

$$V_2 = \frac{Q_2}{C_2} \tag{S9}$$

Substituting Eqs. (S5, S6, S8, S9) into Eq. (S7), we can obtain that:

$$V_{(F)} = \frac{1}{\varepsilon_0 A}\left(\frac{Q_1 x_{(F)}}{\varepsilon_1} + \frac{Q_2 d_{(F)}}{\varepsilon_2}\right) \tag{S10}$$

It can be seen that this result is consistent with the eqn (4), which also reflects the TENG can be regarded as a model of two capacitors connected in series.

From the perspective of mechanical model, the device structure can be simplified to a combination of common mechanical models. For the better analysis of mechanical deformation of our device under the applied force, it can be considered as the series model of simple supported beam and parallel springs as shown in Fig. S9a and b. The upper electrode consists of an aluminum shielding layer, a PET film with coated graphene. In this case, the deformation of the upper electrode could be regarded as simple supported beam system. According to the engineering mechanics theory, the maximum deformation of the upper electrode is:

$$\Delta x = \frac{FL^3}{48YI} \tag{S11}$$

where L is the length of the upper electrode, Y is the Younge's modulus of the upper electrode and I is the moment of inertia.

The force would be transferred from spacer to dielectric layer which could be considered as a parallel muti-springs system. The deformation of the dielectric layer could be calculated by:

$$\Delta d = \frac{F}{k} \tag{S12}$$

where k is the equivalent stiffness coefficient of the parallel muti-springs system.

In this case, the height of the gap layer and dielectric layer under the applied force are:

$$x_{(F)} = x_0 - \Delta x \tag{S13}$$

$$d_{(F)} = d_0 - \Delta d \qquad \text{(S14)}$$

where x_0 and d_0 is the initial height of the gap layer and the dielectric layer.

Substituting Eqs. (S11-S14) into Eq. (S10), we can obtain that:

$$V_{(F)} = \frac{Q_1}{\varepsilon_0 \varepsilon_1 A}(x_0 - \frac{FL3}{48YI}) + \frac{Q_2}{\varepsilon_0 \varepsilon_2 A}\left(d_0 - \frac{F}{k}\right) \qquad \text{(S15)}$$

In this work, to study the relationship of Y and k between the sensitivity, the transferred charge Q_1 and Q_2, dielectric constant ε_0, ε_1 and ε_2, the area of the upper electrode A, the length of upper electrode L and its inertia moment I can be considered as constants. Therefore, the derivative of voltage can be simplified as:

$$\frac{\partial V_{(F)}}{\partial F} = C \cdot \frac{1}{Y} + C_0 \cdot \frac{1}{k} \qquad \text{(S16)}$$

On the other hand, the initial voltage V_0 also can be seen as a constant. Therefore, the sensitivity of the FTEP can be simplified as:

$$S = \frac{\partial V_{(F)}}{\partial P} = \frac{\frac{\partial V_{(F)}}{V_0}}{\frac{\partial F}{A}} = \frac{\partial V_{(F)}}{\partial F} \cdot \frac{A}{V_0} = C_1 \cdot \frac{1}{Y} + C_2 \cdot \frac{1}{k} \qquad \text{(S17)}$$

From the above analysis, it can be concluded that the lower Younge's modulus of the upper electrode and the equivalent stiffness coefficient of the porous dielectric layer, the higher sensitivity of the TENG sensor.

Supplementary Figures and Tables

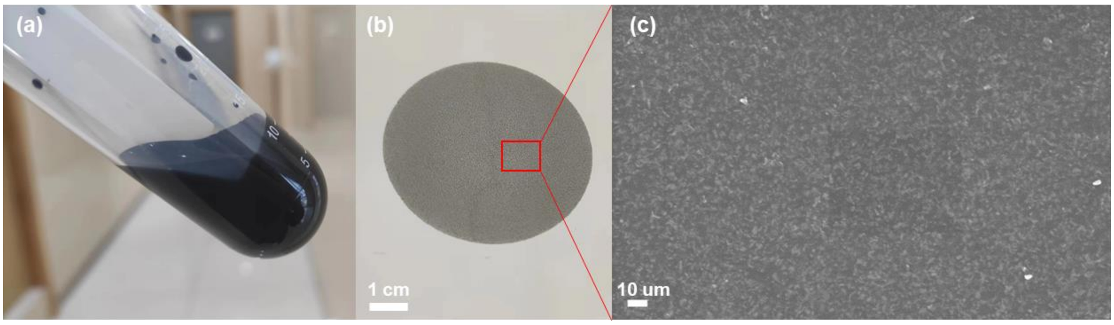

Fig. S1 Photographs of graphene electrodes. a Picture of conductive graphene ink. **b, c** Optical picture and SEM picture of graphene electrode after the printing is completed

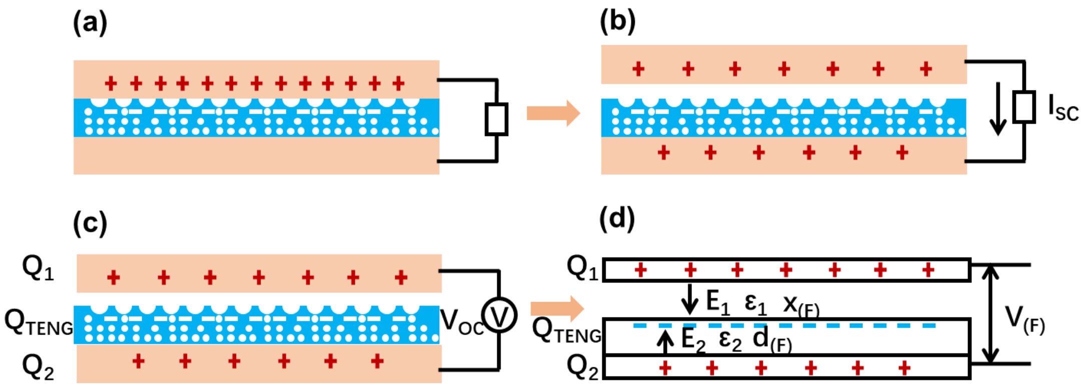

Fig. S2 Schematic illustration of the electricity-generating process and its physical model. a Initial contact state. **b** Short-circuit state. **c** Open-circuit state and **d** its physical model

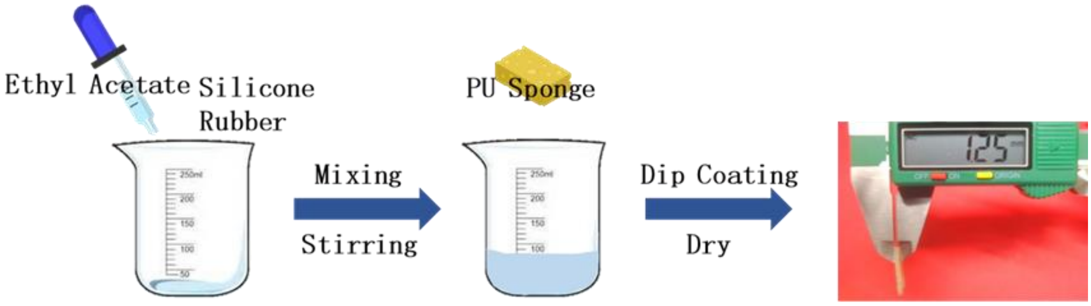

Fig. S3 Fabrication processes of the porous dielectric layer

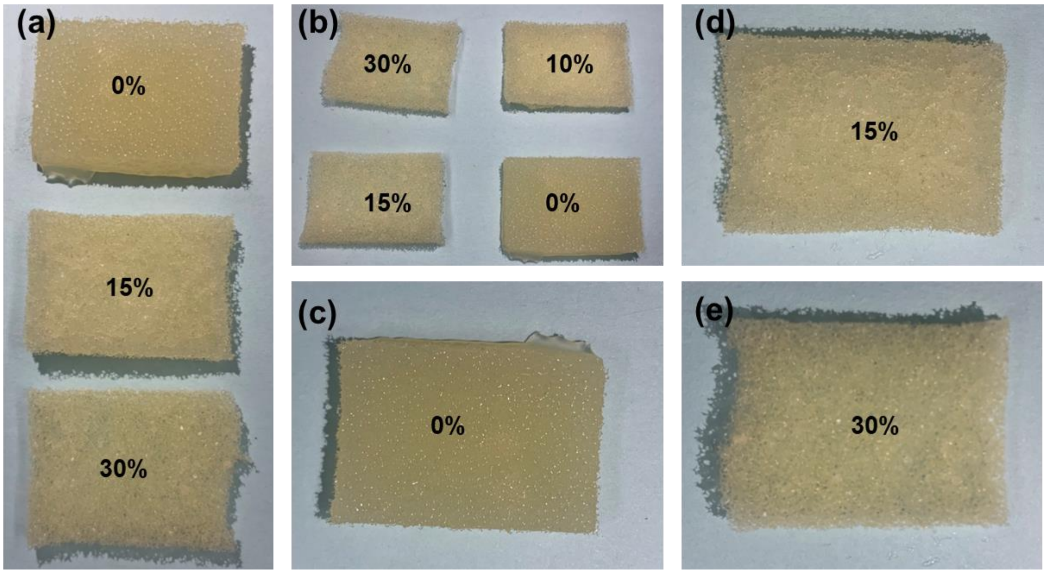

Fig. S4 Optical photographs of PU sponge with various proportions of silicone rubber

From the pictures in Fig. S4, it can be seen clearly that the silicone rubber enters the pores and fills the pore structure of the sponge when the sponge framework is impregnated with different concentrations of silicone rubber solution. The PU dielectric layer with lower dilution ratio than 10% can be seen as a solid state and the layer with higher dilution ratio than 30% is a porous state.

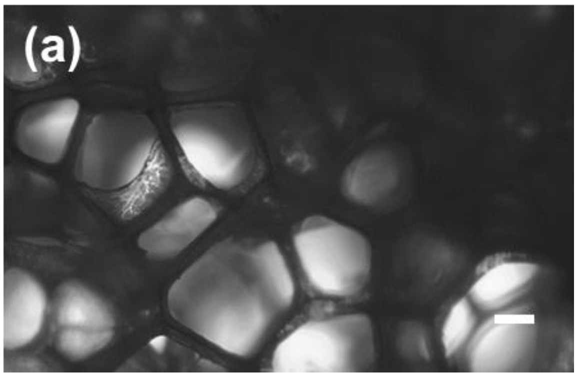

Fig. S5 Optical picture of PU sponge with 30% silicone rubber dilution

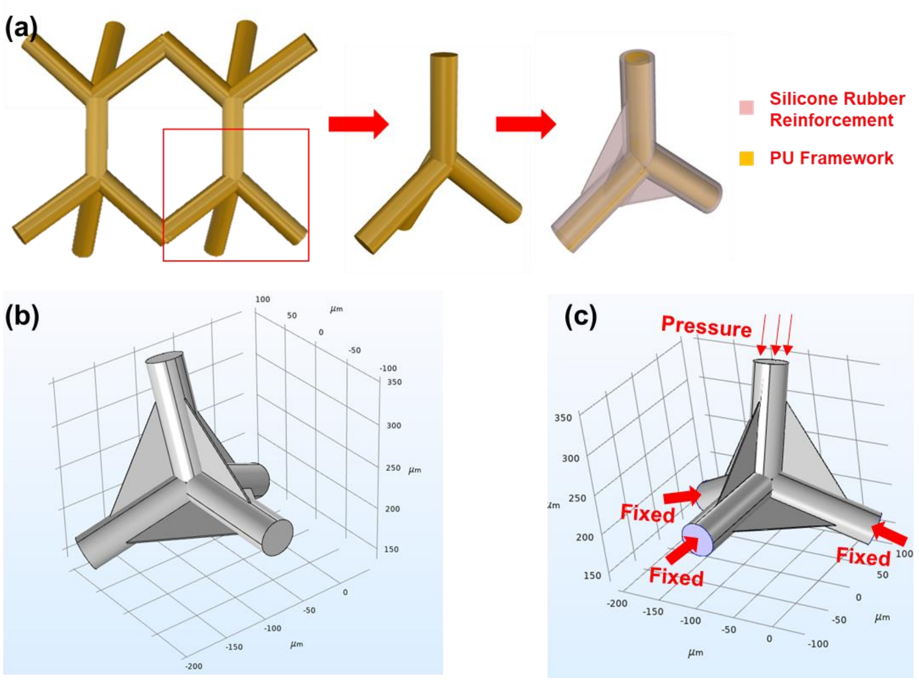

Fig. S6 Finite Element Analysis (FEA) of porous-reinforcement microstructure. a Simplified process of sponge framework model. **b** Simplified model in COMSOL software. **c** Boundary conditions for FEA

As demonstrated in Fig. S6a, the sponge skeleton structure can be regarded as several tetrahedral skeletons connected at the beginning and end. The simplified model, a single tetrahedral skeleton, has been constructed by SolidWorks software for FEA since the sponge skeleton structure is symmetry. The length of the skeleton is 150 μm and the diameter is 40 μm. The volume of reinforcement structure with a thickness of 10 um on the skeleton is a variant. Figure S6b is the simplified model imported from SolidWorks to COMSOL Multiphysics for FEA. The internal skeleton material and external reinforcement material have been set as PU and silicone rubber respectively. Figure S6c shows the boundary conditions for the analysis process. The fixed constraints on the three marked ends of the skeleton have been set, and a uniform load of 1 kPa at the upper end has been applied.

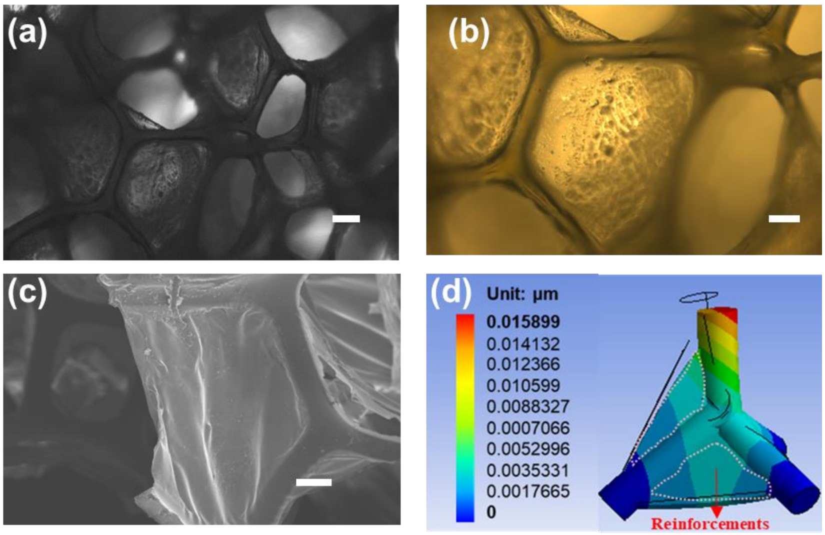

Fig. S7 Partial enlargement pictures of PU sponge with various proportions of silicone rubber. a PU sponge with 15% silicone rubber dilution, **b** its enlarged optical image and **c** its enlarged SEM image. The scale bar is 100 μm. **d** FEA result of PU sponge with 15% silicone rubber dilution

Figure S7a-c show optical magnification and SEM images of the porous dielectric layer at a dilution concentration of 15%, from which it can be seen that the cured silicone rubber content is high and almost fills most of the pores. The contribution of this silicone rubber to the mechanical properties of the porous dielectric layer is mainly due to the reinforcing role played by the silicone rubber in the pores.

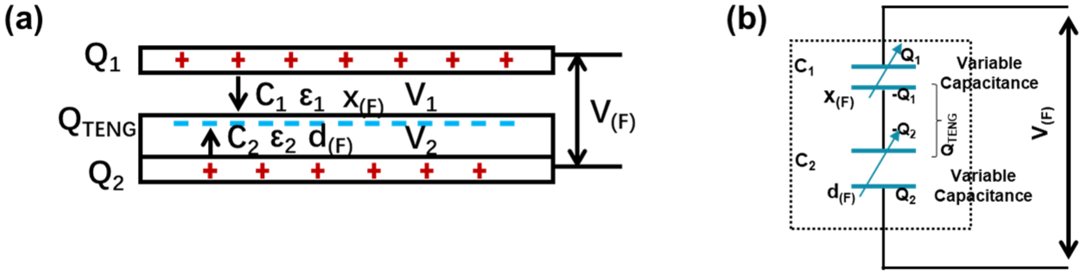

Fig. S8 a Electrical model of the FTEP and **b** its equivalent capacitance model

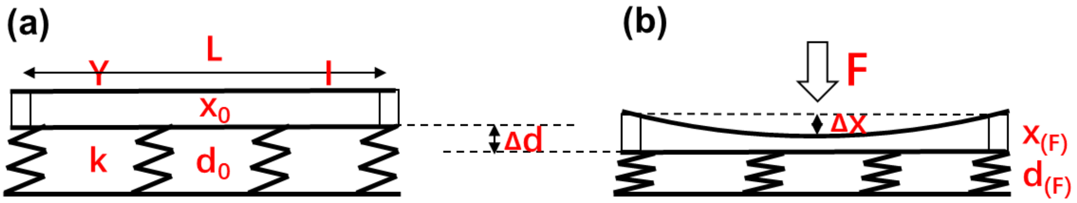

Fig. S9 Mechanical models of the FTEP at **a** initial state and **b** pressing state

For the better analysis of mechanical deformation of our device under the applied force, it can be considered as the series model of simple supported beam and springs as shown in Fig. S7. The upper electrode consists of an aluminum shielding layer, a PET film with coated graphene. In this case, the deformation of the upper electrode could be regarded as simple supported beam system.

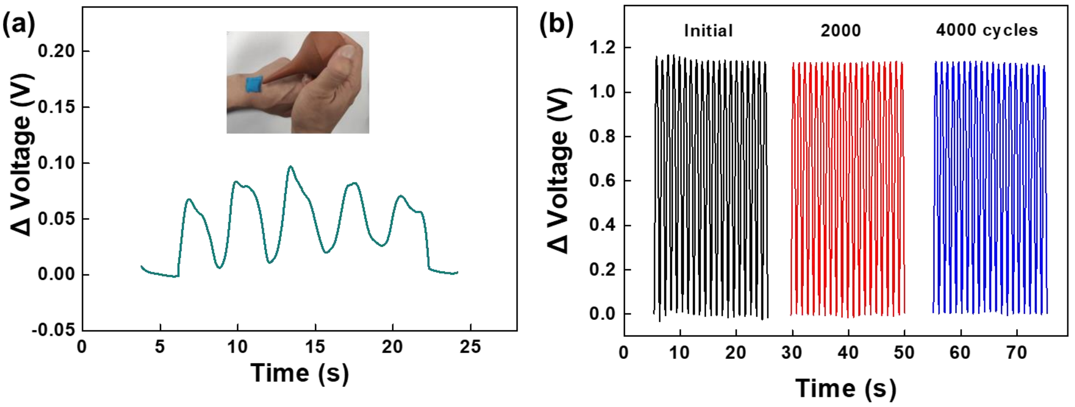

Fig. S10 Tiny pressure detection and stability testing of FTEP. a Voltage signals of FTEP generated by air flow from compressed elastic ball. **b** Stability test for pressure sensing of FTEP with continuous loading and unloading a cyclic external pressure of 5 kPa for 4000 cycles

Figure S8a shows the extraordinary performance for tiny pressure detection and Fig. S8b shows the outstanding repeatability and stability of our sensor through the stability test after 4000 loading/unloading cycles.

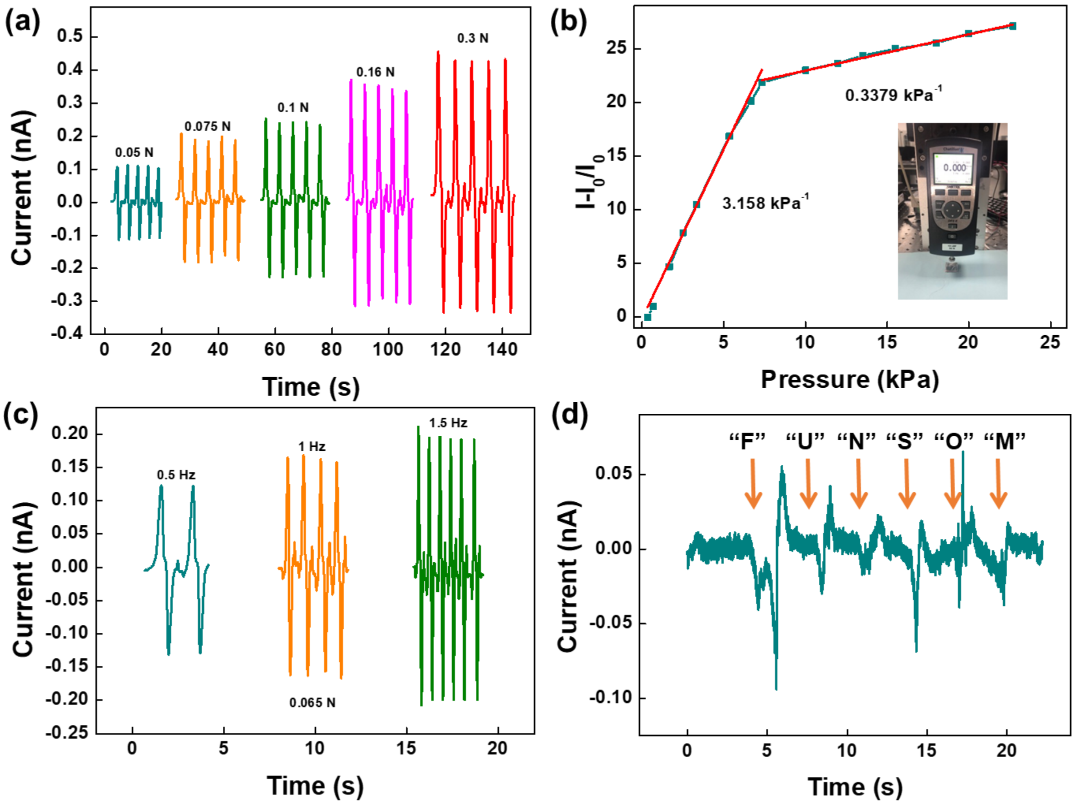

Fig. S11 Dynamic pressure sensing performance of the FTEP through measurement of short-circuit current. a Short-circuit current of the FTEP under various external pressure. **b** Relationship and linear fitting between the relative variations of current and the pressure applied on FTEP. **c** Measurement of current with a constant force of 0.065 N at variable frequencies. **d** Recorded current signals generated by people

The measured short-circuit current with variable pressure under the same loading rate were displayed in Fig. S11a, showing a clearly increasing trend with elevated pressures. Figure S11b is the sensitivity curve based on short-circuit current for FTEP, and it can be clearly seen that the plot also can be divided into two regions based on the difference in sensitivity. In the low-pressure range (0-8 kPa), the sensitivity of FTEP is up to 3.158 kPa^{-1} and still maintains 0.3379 kPa^{-1} in the high-pressure range (8-25 kPa). Furthermore, the performance of FTEP for variable applied frequency under the same force was investigated. As demonstrated in Fig. S11c, the current increases steadily with the elevated frequency form 0.5 to 1.5 Hz under the same applied force 0.065 N, which reveals that the FTEP can detect the loading frequency effectively. And also, the FTEP can detect clearly the voice vibration as demonstrated in Fig. S11d.

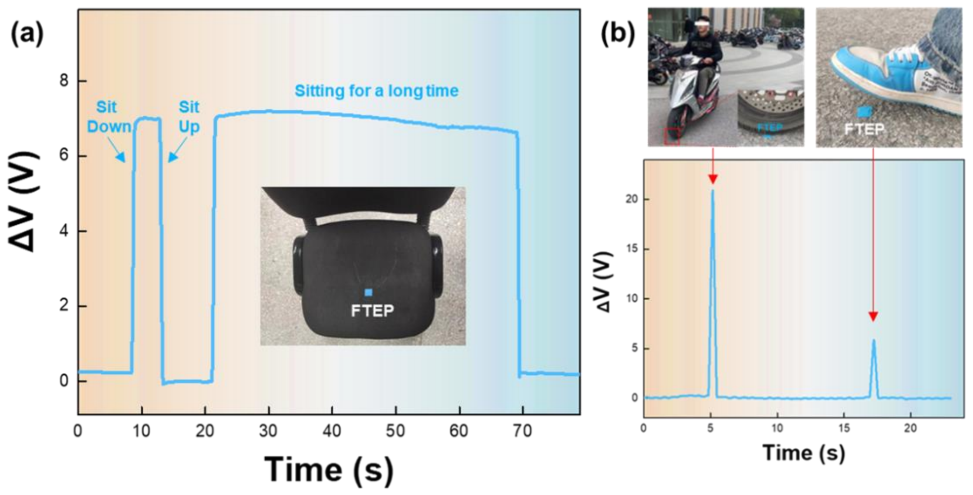

Fig. S12 Sensing performance of the FTEP for the large pressure. a Voltage variation of the sitting state and sedentary. **b** Signal difference between the motorcycle weight and human body weight

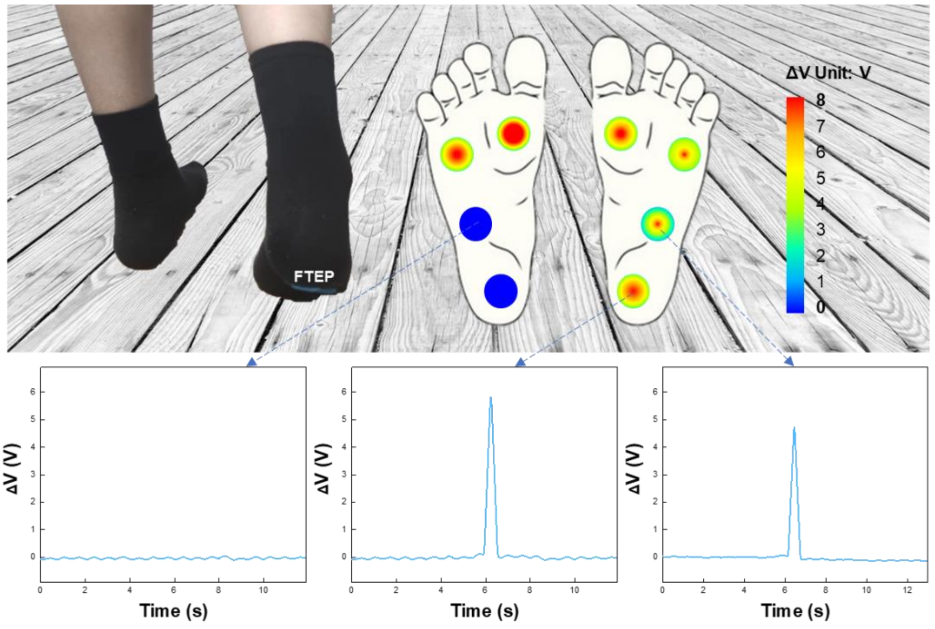

Fig. S13 Plantar pressure distribution recorded by the 2×2 pressure sensor array based on the FTEP

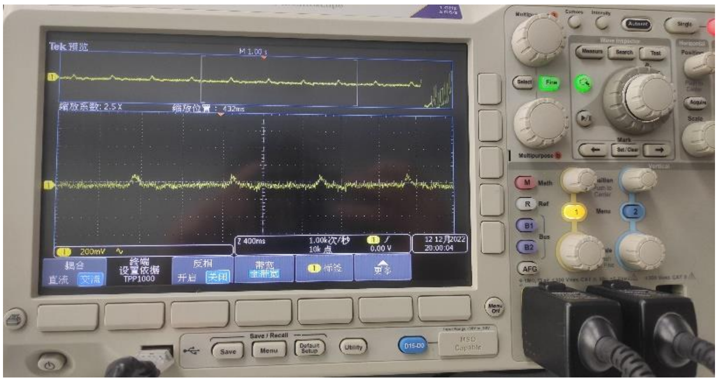

Fig. S14 Pulse wave signals monitored by the oscilloscope

Table S1 Performance comparison of typical triboelectric pressure sensors

**Materials**	**Sensor structure**	**Sensitivity**		**Detection range**	**Refs.**
Ag NWs-Ag@C@PDMS	Micro-porous	<0.7 kPa<br>0.7-10.3 kPa<br>10.3-18 kPa	90.95 V/kPa<br>5.49 V/kPa<br>0.46 V/kPa	0-18 kPa	[S4]
Silicone Rubber-Conductive Sponge	Micro-porous	11.3-38.7 kPa	0.42 kPa^{-1}	11.3-38.7 kPa	[S5]
PDMS-P(VDF-TrFE)	Nano-porous and Micro-ridge	<20 kPa<br>20-100 kPa	0.55 V/kPa<br>0.2 V/kPa	0-100 kPa	[S6]
PTFE-PAM Hydrogel	Micro-pyramid	<0.3 kPa<br>0.3-2 kPa	1.4 kPa^{-1}<br>0.23 kPa^{-1}	0-2 kPa	[S7]
PTFE-Ferrofluid	Micro-cone	<2.5 kPa<br>2.5-35 kPa	21.48 kPa^{-1}<br>1.14 kPa^{-1}	0-35 kPa	[S8]
Silicone Rubber-Graphene	Porous-reinforcement	<5 kPa<br>5-25 kPa	5.93 kPa^{-1}<br>0.21 kPa^{-1}	0-25 kPa	This work

Supplementary Movies

Movie S1 The responses of FTEP for the tiny pressure.

Movie S2 Pulse wave and heart rate detection.

Movie S3 The wearable Internet of Healthcare (IoH) system.

Supplementary References

[S1] S. Niu, S. Wang, L. Lin, Y. Liu, Y. S. Zhou et al., Theoretical Study of Contact-Mode Triboelectric Nanogenerators as an Effective Power Source. Energy Environ. Sci. **6**(12), 3576–3583 (2013). https://doi.org/10.1039/c3ee42571a

[S2] R. Hinchet, A. Ghaffarinejad, Y. Lu, J. Y. Hasani, S.-W. Kim et al., Understanding and modeling of triboelectric-electret nanogenerator. Nano Energy **47**, 401-409 (2018). https://doi.org/10.1016/j.nanoen.2018.02.030

[S3] S. Lee, J.-W. Park, Fingerprint-inspired triboelectric nanogenerator with a geometrically asymmetric electrode design for a self-powered dynamic pressure

sensor. Nano Energy **101**, 107546 (2022). https://doi.org/10.1016/j.nanoen.2022.107546

[S4] Z. Peng, X. Xiao, J. Song, A. Libanori, C. Lee et al., Improving Relative Permittivity and Suppressing Dielectric Loss of Triboelectric Layers for High-Performance Wearable Electricity Generation. ACS Nano **16**, 20251–20262 (2022). https://doi.org/10.1021/acsnano.2c05820

[S5] J. Chen, B. Chen, K. Han, W. Tang, Z. L. Wang, A Triboelectric Nanogenerator as a Self-Powered Sensor for a Soft–Rigid Hybrid Actuator. Adv. Mater. Technol. **4**, 1900337 (2019). https://doi.org/10.1002/admt.201900337

[S6] M. Ha, S. Lim, S. Cho, Y. Lee, S. Na et al., Skin-Inspired Hierarchical Polymer Architectures with Gradient Stiffness for Spacer-Free, Ultrathin, and Highly Sensitive Triboelectric Sensors. ACS Nano **12**, 3964–3974 (2018). https://doi.org/10.1021/acsnano.8b01557

[S7] L. Jia, Z. H. Guo, L. Li, C. Pan, P. Zhang et al., Electricity Generation and Self-Powered Sensing Enabled by Dynamic Electric Double Layer at Hydrogel-Dielectric Elastomer Interfaces. ACS Nano **15**, 19651–19660 (2021). https://doi.org/10.1021/acsnano.1c06950

[S8] J. Liu, Z. Wen, H. Lei, Z. Gao, and X. Sun, A Liquid–Solid Interface-Based Triboelectric Tactile Sensor with Ultrahigh Sensitivity of 21.48 kPa^{-1}. Nano-Micro Lett. **14**, 88 (2022). https://doi.org/10.1007/s40820-022-00831-7
